# Differentiation of Cocaine-Induced Midline Destructive Lesions from ANCA-Associated Vasculitis

**Published:** 2018-09

**Authors:** Alireza Mirzaei, Mozhdeh Zabihiyeganeh, Ala Haqiqi

**Affiliations:** 1 *Bone and Joint Reconstruction Research Center, Shafa Orthopedic Hospital, Iran University of Medical Sciences, Tehran, Iran.*; 2 *Department of General Medicine, St. George’s University Hospitals NHS Foundation Trust, London, UK.*

**Keywords:** Anti-neutrophil cytoplasmic antibody, Cocaine, Granulomatosis

## Abstract

**Introduction:**

Cocaine-induced midline destructive lesions (CIMDL) are complications of regular nasal cocaine inhalation. CIMDL can mimic systemic diseases with positive anti-neutrophil cytoplasmic antibodies (ANCA), such as granulomatosis with polyangiitis (GPA).

**Case Report:**

In this article, we describe the case of a young woman who presented with nasal perforation induced by cocaine, along with positive perinuclear ANCA test (proteinase 3 antigen), misdiagnosed as limited GPA. The patient was treated with immunosuppressive therapy, which partially improved her symptoms. Admittance of cocaine use aided in the diagnosis of CIMDL. This patient was advised to stop cocaine use. Three-month follow-up revealed no further complications.

**Conclusion:**

Considering the seropositivity of ANCA in both CIMDL and GPA, early diagnosis of CIMDL and its differentiation from GPA is crucial, and clinicians play an important role in this regard. Lack of distinct histologic characteristics of vasculitis or unresponsiveness to standard therapeutic regimens may favor the diagnosis of CIMDL syndrome. It is crucial to recognize that these conditions may have similar presentations, so that undesired and potentially toxic treatments can be prevented.

## Introduction

The prevalence of cocaine use is growing worldwide (1,2). However, due to its high price, it is not a widely used recreational drug in third-world countries, and as a result, its complications are usually difficult to distinguish from other diseases in those countries because its use is rare. Also, presentation to a healthcare professional is rare due to significant cultural taboos and fear of rejection.

Nasal and paranasal tissue damage and necrosis are sequelae of frequent and regular cocaine nasal inhalation. This frequent inhalation may result in an inflamed and ulcerated nasal mucosa and perforated septum. This complication is termed “cocaine-induced midline destructive lesion (CIMDL)” (1,3). Prevalence of CIMDL is 4.8% among cocaine users (4). CIMDL mimics the clinical manifestations of systemic diseases with positive anti-neutrophil cytoplasmic antibodies (ANCA), such as small-vessel vasculitis, including granulomatosis with polyangiitis (GPA) (5-7). Interestingly, ANCA has been detected in a significant number of CIMDL cases in which clinical presentation of lesions was indistinguishable from GPA limited to the upper respiratory tract (5,8,9).

The pathogenesis of CIMDL is largely unknown, but inflammatory, infective, pro-apoptotic and autoimmune mechanisms have been implicated (4). One hypothesis is that longstanding cocaine usage could cause mucosa and perichondrium progressive destruction via a direct vasoconstrictor impact (2,10). Necrotic septal cartilage due to ischemia and septal perforation, which resembles the upper airway clinical pictures of GPA, would thus ensue.

Differentiating CIMDL from limited GPA is challenging, especially when the clinical history of cocaine abuse is vague and its use is not common.

Here we describe the case of a young lady in Iran who presented with cocaine-induced nasal perforation and positive ANCA test, primarily misdiagnosed as ANCA-associated vasculitis because of the patient’s denial of substance dependence.

## Case Report

A 30-year-old woman was referred to our clinic with a 2-month history of epistaxis and necrotizing lesions of her nose with a background of chronic sinusitis and rhinorrhea. Ear, nose, and throat (ENT) examination of the patient indicated a defect in her nasal septum.

**Fig1:**
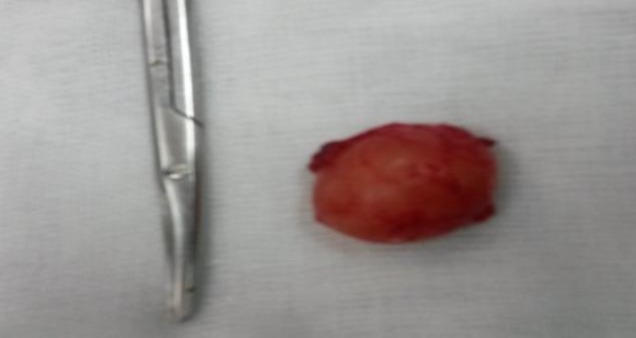
CT scan of sinus revealing a thickness in the right maxillary sinus and a defect in the anterior aspect of the nasal septum

**Fig2:**
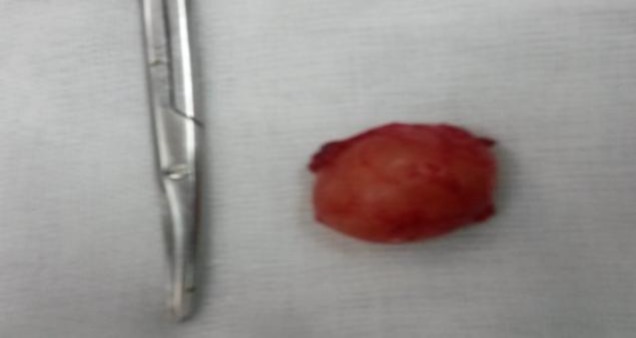
Histopathology slides of the nasal biopsy specimens revealed ulceration of the epithelium with granulation tissue formation. (A) Severe infiltration of mixed inflammatory cells in submucosa with infiltration into vascular wall (arrow) (x200). (B)Ulceration of nasal mucosa (arrow) associated with fibrin exudate material and severe mixed inflammation of submucosa (x200).

The coronal and axial cut of computed tomography (CT) reconstructions of nasal soft tissues and paranasal sinuses revealed mucosal thickening in the right maxillary sinus and a defect in the anterior aspect of the nasal septum with an approximate diameter of 13 mm (Fig.1). The nasal septum biopsy revealed respiratory mucosa with ulceration, acute inflammation, and granulation tissue formation (Fig.2).

Kidney function and chest X-ray were normal. Laboratory findings were leukocyte count 13,600 per μl (normal range 4,500 to 11,000), which was mildly elevated; hemoglobin 13 g/dl; platelets 373,000/mm³; high erythrocyte sedimentation rate (ESR) 35 mm/h (normal range up to 20); elevated C-reactive protein (CRP) 45 mg/L (normal range up to 6); perinuclear ANCA (p-ANCA) 1/320 (normal range up to 1/10) with positive antigen-specific ANCA directed against proteinase 3 (PR3) and negative myeloperoxidase (MPO), cytoplasmic ANCA (c-ANCA), antinuclear antibody (ANA) and rheumatoid factor. All tests for HIV virus, hepatitis C and hepatitis B viruses, FTA-ABS and venereal disease research laboratory (VDRL) were negative. Purified protein derivative (PPD) test was not reactive. Leishmaniasis and blastomycosis serology were also negative.

When asked, the patient denied being a drug abuser. Thus, the combination of clinical, serologic and histologic findings, including ulceration, inflammation, and granulation tissue formation (Fig.2) led to the diagnosis of ANCA-associated vasculitis, most likely limited GPA.

Her treatment with prednisolone (50 mg/day), sulfamethoxazole and trimethoprim was started, followed by adding methotrexate (15 mg/week). After 3 months, with no evidence of improvement clinically or serologically, and considerable weight gain due to the high-dose steroid, rituximab was administered to the patient, but with only a minor improvement in the symptoms. At this point, the patient admitted the ongoing nasal use of cocaine. Subsequently, the causative effect of cocaine was suspected, and the patient was advised to stop cocaine abuse. During 3 months of further clinical follow-ups, no additional new problems related to CIMDL were identified. Written informed consent was obtained from the patient in order to publish her case.

## Discussion

We are witnessing a remarkable rise in cocaine use worldwide at this time. Consequently, destructive mid-facial lesions caused by CIMDL, mimicking the clinical picture of other diseases, is likely to become a more common scenario (1,2). Nonetheless, because of the high price of cocaine, it is still not commonly in use among drug users in low-socioeconomic areas of the world. Overlap of clinical signs and symptoms means that complications with this drug can be easily misdiagnosed with those of other diseases. As a result, the positive result of the ANCA test makes the differentiation of this condition from middle- and small-vessel vasculitis, such as limited GPA, more challenging.

Nasal involvement is frequently seen in many rheumatologic illnesses. These signs are helpful to establish a diagnosis. Therefore, these patients’ assessments and investigations should include a multidisciplinary approach, such as a rigorous history and thorough clinical examination.

A study by Armengot et al. elucidated that GPA has the highest level of sinonasal involvement among the systemic vacuities (6). GPA is a systemic disease characterized by granulomatous inflammation and necrosis of the upper and lower respiratory tract, a vasculitis which is mainly noted in small- and medium-size vessels, with focal segmental or proliferative glomerulosclerosis (11).GPA can present at any age; however, it is most prevalent around the age of 40 (6). It is interesting to note that, as in our case, the majority of previous cases of CIMDL were in women from a relatively young age group (10). This could suggest that female users may be more susceptible to the nasal complications.

Nasal manifestations are the presenting signs in around 50–90% of GPA cases (12), and ANCAs are present in up to 83% of cases of limited GPA (13). It is of particular note that according to the study by Wiesner et al., 84% of the CIMDL patients were found to have a positive ANCA test (9). A distinct p-ANCA pattern targeting elastase or other nonspecific antigens are shown in CIMDL (14). All these findings together could potentially complicate making a differential diagnosis.

Although the precise pathophysiology of ANCA abnormalities is not completely known, ANCA positivity could be correlated with the presence of *Staph aureus*. It has been revealed that *Staph aureus* nasal carriers are linked with greater rates of relapse in GPA (15). A significant number of CIMDL patients are also carrying Staph aureus in their nose and cultures of nasal tissue grow Staph aureus (2,16).

As mentioned above, cocaine-associated mucosal destruction is multifactorial. Focal mucosal vasoconstriction is believed to play a leading role (2-4,10). Furthermore, although invasive fungal rhinosinusitis may be a serious complication of intranasal cocaine abuse (2), it was not detected in this patient. It is imperative to diagnose correctly whether the lesions stem from autoimmune vasculitis or cocaine abuse, because the management plans and clinical pathways are different (6). Clinical, analytical, and histopathological findings can distinguish between these two conditions (6).

Spontaneously resolving epistaxis, rhinorrhea, and scabs are the most common symptoms, as were also seen in this patient (10,12). Differentiation of these manifestations from typical GPA is not always easy. In CIMDL, the sinonasal region is mostly affected without the involvement of the other parts (1).

Histopathologically, while acute inflammatory and chronic perivascular infiltrates are seen in a few CIMDL patients, the salient feature of this condition is extensive necrosis. GPA-associated features (granulomas, giant multinucleated cells, leukocytoclasia, or fibrinoid changes) are rarely observed in CIMDL (2). Granulation tissue formation along with no typical granuloma, as seen in our case, could help the correct diagnosis of similar cases. 

Another form of the cocaine-induced syndrome is levamisole-induced vasculitis. In total, 70% of the available batches of cocaine are contaminated with levamisole (4), which is currently used as a veterinary anthelmintic medication and appears to be a ubiquitous adulterant associated with cocaine abuse (17). This condition has a diverse range of auto-immunoglobulin findings, including p-ANCA and c-ANCA. The probable mechanism of ANCA positivity in CIMDL could be nasal carriage of *Staph aureus* that can trigger the autoimmune system (15). The fact that around 70% of cocaine is contaminated with levamisole may also partly account for the ANCA positivity in this syndrome (7). Considering its more systemic manifestations compared with CIMDL, its differentiation from autoimmune conditions like GPA and other small-vessel vasculitis might be even more challenging (18). While we did not have any evidence of levamisole-adulterated cocaine use in our case, when treating a patient with features of systemic vasculitis resistant to treatment, it would be worth checking whether the cocaine used by the patient contains levamisole. The optimal therapy for CIMDLis aggressive immunosuppressive therapy, including corticosteroid, cyclophosphamide, mycophenole mofetil, and methotrexate, which are options in those with the substantial inflammatory disease, or in cases showing no improvement following the cessation of cocaine use (1).

Awareness of ANCA positivity along with clinical manifestations similar to vasculitis in CIMDL could lead to a correct diagnosis of CIMDL, especially in regions with lower use or availability of cocaine.

In our case, the presence of 1) a nasal defect along with ulceration of the epithelium with granulation tissue formation; 2) severe infiltration of mixed inflammatory cells in submucosa with infiltration into vascular wall in histopathologic examination; 3) the positive ANCA test (confirmed by both ELISA and indirect immunofluorescence methods), and 4) elevated inflammatory markers, led to the misdiagnosis of ANCA-associated vasculitis. In addition to these, the patient’s denial of drug abuse with cocaine contributed to the clinicians’ wrong identification of the underlying cause of the clinical and para-clinical features.

A urine test in cases of suspected cocaine abuse may be a useful tool to determine whether these clinical manifestations and treatment failure are in keeping with ongoing cocaine abuse. Benzoylecgonine is a cocaine metabolite, which can be detected in urine for 2 weeks following use (19).

## Conclusion

Differentiation between ANCA-associated primary vasculitis and CIMDL is challenging. This is due to their similar clinical, serological, and histological manifestations. Despite the similarities of CIMDL with diseases like Wegener's granulomatosis, the presence of nasal septum defect, the lack of distinct vasculitis histologic findings, and unresponsiveness to standard therapeutic regimen may favor the diagnosis of CIMDL syndrome. In order to avoid unnecessary harmful immunosuppressant therapy, a thorough history-taking and exploration of intranasal insufflations of cocaine play an important role in differentiating these conditions. While immunosuppressive therapy is likely to help in the management of this condition and to limit the progressive damage, stopping cocaine abuse is the mainstay of treatment.
